# Inconsistency in Abnormal Brain Activity across Cohorts of ADHD-200 in Children with Attention Deficit Hyperactivity Disorder

**DOI:** 10.3389/fnins.2017.00320

**Published:** 2017-06-06

**Authors:** Jian-Bao Wang, Li-Jun Zheng, Qing-Jiu Cao, Yu-Feng Wang, Li Sun, Yu-Feng Zang, Hang Zhang

**Affiliations:** ^1^Center for Cognition and Brain Disorders and the Affiliated Hospital, Hangzhou Normal UniversityHangzhou, China; ^2^Zhejiang Key Laboratory for Research in Assessment of Cognitive ImpairmentsHangzhou, China; ^3^Institutes of Psychological Sciences, College of Education, Hangzhou Normal UniversityHangzhou, China; ^4^Institute of Mental Health, The Sixth Hospital, Peking UniversityBeijing, China; ^5^Paul C. Lauterbur Research Centers for Biomedical Imaging, Shenzhen Institutes of Advanced Technology, Chinese Academy of SciencesShenzhen, China

**Keywords:** attention deficit hyperactivity disorder, resting state fMRI, multi-site dataset, ADHD-200, voxel-wise whole-brain analysis

## Abstract

Many papers have shown results from the multi-site dataset of resting-state fMRI (rs-fMRI) in attention deficit hyperactivity disorder (ADHD), a data-sharing project named ADHD-200. However, few studies have illustrated that to what extent the pooled findings were consistent across cohorts. The present study analyzed three voxel-wise whole-brain metrics, i.e., amplitude of low-frequency fluctuation (ALFF), regional homogeneity (ReHo), and degree centrality (DC) based on the pooled dataset as well as individual cohort of ADHD-200. In addition to the conventional frequency band of 0.01–0.08 Hz, sub-frequency bands of 0–0.01, 0.01–0.027, 0.027–0.073, 0.073–0.198, and 0.198–0.25 Hz, were assessed. While the pooled dataset showed abnormal activity in some brain regions, e.g., the bilateral sensorimotor cortices, bilateral cerebellum, and the bilateral lingual gyrus, these results were highly inconsistent across cohorts, even across the three cohorts from the same research center. The standardized effect size was rather small. These findings suggested a high heterogeneity of spontaneous brain activity in ADHD. Future studies based on multi-site large-sample dataset should be performed on pooled data and single cohort data, respectively and the effect size must be shown.

## Introduction

Attention deficit hyperactivity disorder (ADHD) is one of the most common neurodevelopmental disorders in children (Polanczyk et al., 2015). It is a highly heterogeneous disease, involving multiple deficits and multiple neural pathways (Castellanos et al., 2006; Bush, 2010). The complicated pathophysiology of ADHD has been widely investigated through task and resting-state functional magnetic resonance imaging (fMRI) studies. Task-state fMRI studies commonly employed various task paradigms, e.g., Go/No Go (Schulz et al., 2004; Newman et al., 2015), Eriksen Flanker Task (Vaidya et al., 2005; Vasic et al., 2014). These tasks are complicated, and various paradigms did not exhibit consistent results (Cortese et al., 2012). In contrast, resting-state fMRI (rs-fMRI) is easy to be implemented and provides a consistent approach for clinical investigations. Thus, more and more researchers perform rs-fMRI studies on brain disorders, including ADHD.

ADHD-200, as one of the most widely used multi-site MRI dataset of brain disorders, has attracted considerable attention from the ADHD research community. This dataset released by ADHD-200 consortium contains ten independent cohorts from eight different sites (ADHD-200-Consortium, 2012). These cohorts provide rs-fMRI and anatomical MRI data of both ADHD and typically developing children (TDC), about 776 participants in total. ADHD-200 facilitated the investigation of the neural basis of ADHD, and about 30 studies based on this dataset have been published according to PubMed (e.g., Tomasi and Volkow, 2012; Elton et al., 2014; Sripada et al., 2014; Carmona et al., 2015).

Most studies on ADHD-200 pooled data of cohorts and explored the abnormal brain activity for ADHD. Increasing number of these studies were reported in recent years. For example, Mills et al. (2012) pooled data of Brown University (BU), Peking University (PKU), Kennedy Krieger Institute (KKI), and New York University (NYU) together and observed increased connection between the medial and anterior dorsal thalamus and the basal ganglia in ADHD (Mills et al., 2012). Pooling data of PKU, NYU together, Zhang et al. (2014) found affected brain regions in ADHD mainly located in the orbito-frontal cortex, inferior/superior frontal gyrus, anterior cingulate gyrus, and calcarine cortex (Zhang et al., 2014). Pooling cohorts together facilitated the establishment of a large sample size and tended to provide very positive results. However, to what extent the pooled results are consistent across individual cohorts remains unknown. To the best of our knowledge, only one study on ADHD-200 dataset answered this question (Cai et al., 2015). They found that ADHD group of cohorts NYU, PKU, and OHSU consistently showed decreased network-interaction among the salience network (SN), central executive network (CEN), and default mode network (DMN). Notably, the network analysis could not indicate the exact aberrant brain regions for ADHD, and it remains unclear whether findings of the local brain regions for ADHD are consistent across cohorts or not.

The present study aimed to examine the consistency of abnormal local brain regions across cohorts of ADHD-200. Specifically, we analyzed three voxel-wise whole-brain metrics, i.e., amplitude of low-frequency fluctuation (ALFF) (Zang et al., 2007), regional homogeneity (ReHo) (Zang et al., 2004), and degree centrality (DC) (Buckner et al., 2009). Importantly, the analytic processes of these kinds of methods are very similar across studies, and hence facilitate the coordinate-based meta-analysis (CB-meta) which helps to find regions of consistent activity across fMRI studies (Bartra et al., 2013; Herz et al., 2014; Iwabuchi et al., 2015). Analysis of these metrics is often performed at the frequency band of 0.01–0.08 Hz which has been widely used in rs-fMRI studies. In addition to this conventional band, rs-fMRI signals at some sub-frequency bands can also be modulated by different resting state (e.g., eyes closed and eyes open; Yuan et al., 2014) as well as by disease (e.g., chronic pain; Malinen et al., 2010; Otti et al., 2013). These sub-frequency bands, i.e., Slow-6 (<0.01 Hz; Lv et al., 2013; Zhang et al., 2015a), Slow-5 (0.01–0.027 Hz), Slow-4 (0.027–0.073 Hz; Zuo et al., 2010; Han et al., 2011; Zhang et al., 2013), Slow-3 (0.073–0.198 Hz), and Slow-2 (0.198–0.25 Hz; Wang et al., 2015), were also investigated in the present study in order to obtain more information through the frequency-dependent characteristic.

## Methods and materials

### Subjects and data acquisition

The data we used in this study is publicly available from the ADHD-200 Consortium (http://fcon_1000.projects.nitrc.org/indi/adhd200/). The ADHD-200 dataset contains both functional and anatomical MRI data contributed by eight institutions. Each cohort was approved by the research ethics review boards of each institution. Signed informed consent was obtained from all participants or their legal guardian before participation.

**Figure 1:**
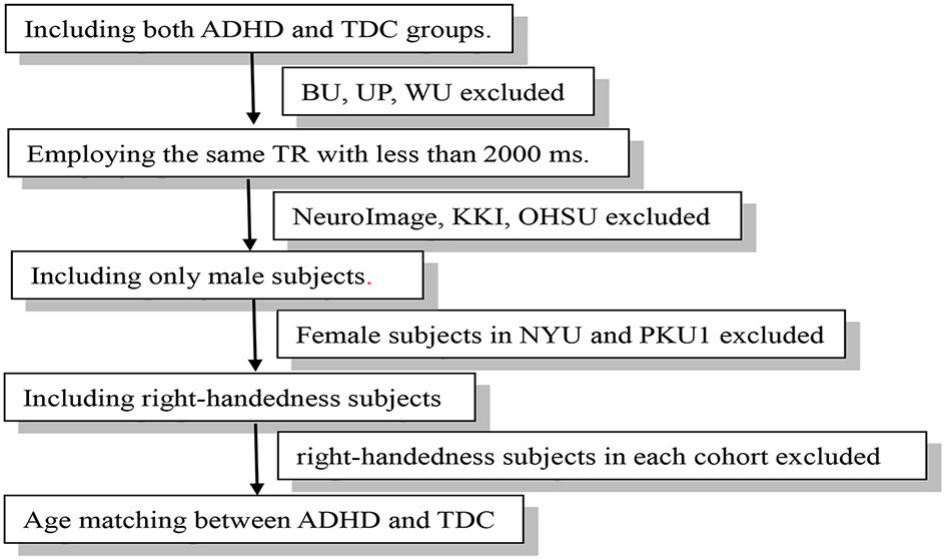
Flow-chart of data exclusion.

We first selected the data cohorts according to the following criteria: (1) Including both ADHD and TDC groups. So the data from the BU, University of Pittsburgh and, Washington University were excluded; (2) Employing the same TR with <2,000 ms across the cohort. According to this criterions, data from NeuroImage (TR = 1,960 ms), KKI (TR = 2,500 ms), and OHSU (TR = 2,500 ms) were excluded. Then, the NYU, PKU1, PKU2, and PKU3 cohorts were included in our research. The PKU2 and PKU3 cohorts only had male subjects, so the female subjects in NYU and PKU1 cohorts were excluded to remove potential confounding effect of gender to the consistency across cohort. Left-handedness subjects were also excluded for each cohort. After case-by-case matching age between ADHD and TDC, 58 subjects from NYU, 30 from PKU1, 56 from PKU2, and 38 from PKU3 were included in the current study. Demographic information was summarized in Table 1. Flow-chart of data exclusion was shown in Figure 1.

**Table 1:** Demographic information of each cohort in the current study.

	**NYU**		**PKU1**		**PKU2**		**PKU3**	
	**ADHD**	**TDC**	**ADHD**	**TDC**	**ADHD**	**TDC**	**ADHD**	**TDC**
*N*	29	29	15	15	28	28	19	19
Gender (male)	29	29	15	15	28	28	19	19
Age (years)	12.1 ± 2.9	12.2 ± 2.8	11.2 ± 2.3	11.6 ± 1.5	12.7 ± 1.7	11.7 ± 1.8	13.2 ± 1.3	13.3 ± 1.0
IQ	106.1 ± 16.0	115.3 ± 14.3	101.7 ± 12.4	123.0 ± 14.2	111.5 ± 12.7	121.6 ± 12.2	102.7 ± 10.4	111.7 ± 12.7
Subtype (C/I/H)	19/10/0	–	9/6/0	–	16/12/0	–	12/7/0	–

*Data are presented as mean ± SD. C, ADHD –Combined; I, ADHD –Inattentive; H, ADHD -Hyperactive/Impulsive*.

Psychostimulant medications were withheld at least 24 h prior to scanning. The inclusion and exclusion criteria and more detailed demographic characteristics of the participants of the four cohorts can be seen in the http://fcon_1000.projects.nitrc.org/indi/adhd200/. The rs-fMRI data of the four cohorts were from three scanners, with TR of 2 s for all. PKU1 and PKU2 used the same scanner but scanning parameters were slightly different. The detailed parameters were listed in the Supplementary Table 1.

### Data preprocessing

Functional images of each subject were preprocessed by using Data Processing Assistant for Resting-State fMRI (DPARSF) (Chao-Gan and Yu-Feng, 2010) which is based on Statistical Parametric Mapping (SPM8) (http://www.fil.ion.ucl.ac.uk/spm) and Resting-State fMRI Data Analysis Toolkit (Song et al., 2011). Preprocessing was performed as follows: removal of the first ten volumes to avoid signal instability and to get subjects adapted to the scanning noise. Then, the number of time point is 170 at least (NYU), so the first 170 volumes were included for individuals in PKU1, PKU2, and PKU3 considering the comparability across cohorts (Molloy et al., 2014; Carmona et al., 2015). Slice timing correction, image realignment to correct head motion were followed. After individual structural images were segmented after co-registered to functional images, functional images were spatial normalized to Montreal Neurological Institute (MNI) space at 3 mm isotropic voxel resolution applying the unified segmentation parameters. The linear trend, head motion parameter measured by Friston-24 model, white matter (WM), and cerebrospinal fluid (CSF) signals were further regressed out as nuisance covariates. Then, three voxel-wise whole-brain analytic methods, i.e., ALFF, ReHo, and DC, were further used to analyze these preprocessed data.

### ALFF calculation

ALFF is the amplitude of low frequency fluctuations of the blood oxygen level dependent (BOLD) signal of every single voxel (Zuo et al., 2010). ALFF calculation was the same as the procedure in Zang et al. (2007). After preprocessing, the 4D rs-fMRI data of each participant was spatially smoothed with a 6 mm FWHM Gaussian kernel and then, the linear trend was removed from the time course of each voxel. Then, ALFF was calculated for the conventional low frequency band (0.01–0.08 Hz) as well as five sub-bands, i.e., Slow-6 (0–0.01 Hz), Slow-5 (0.01–0.027 Hz), Slow-4 (0.027–0.073 Hz), Slow-3 (0.073–0.198 Hz), and Slow-2 (0.198–0.25 Hz).

### ReHo calculation

ReHo is a voxel-wise measure of the local synchronization of the time courses of nearest neighboring voxels (usually 27 voxels). It was calculated by using Kendall's coefficient of concordance (KCC) as follows:

(1) $$\begin{array}{l} W=\frac{\sum {\left(R_{i}\right)}^{2}-n{\left(\bar{R}\right)}^{2}}{\frac{1}{12}K^{2}\left(n^{2}-n\right)} \end{array}$$

where W is the KCC among given voxels, ranged from 0 to 1; *R*_*i*_ is the sum rank of the *i*th time point; $\bar{R}=\left(\left(n+1\right)K/2\right)$ is the mean of *R*_*i*_'s; *K* is the number of time courses within a measured cluster (27 in the current study); and *n* is the number of ranks. After the removing of linear trend, the time course of each voxel, band-pass filtering was performed for six sub-bands as in ALFF analysis. ReHo was then calculated for each sub-band. The spatial smoothing (FWHM = 6 mm) was performed after ReHo calculation as did in previous studies (Zang et al., 2004).

### DC calculation

Degree centrality (DC) represents the node characteristic of large-scale brain intrinsic connectivity networks by capturing the relationship with the entire brain network in the voxel level (Zuo et al., 2012). We used weighted DC since it provides a more precise centrality characterization of functional brain networks than binary version (Cole et al., 2010). Specifically, after preprocessing, the linear trend of the time course of each voxel was removed, and then band-pass filtering was performed for six sub-bands as in ALFF analysis. The Pearson correlation was performed between the time course of each voxel with that of every other voxel in the entire brain (Buckner et al., 2009). The correlation coefficients with *r* > 0.2 were summed up for each voxel and then a weighted DC was obtained for each voxel. 0.2 was used as threshold to eliminate counting voxels that had low temporal correlation and it has been proved that different threshold selections did not qualitatively change the results (Buckner et al., 2009). As did in ReHo calculation, spatial smoothing may introduce possible artificial local correlations, we performed spatial smoothing (FWHM = 6 mm) after DC calculation as did elsewhere as follows (Zuo et al., 2012):

(2) $$\begin{array}{l} \;D=\sum a_{ij}\\ \text{Where }j=1\ldots N,i\neq j,a_{ij}=\{\begin{array}{c} 0,a_{ij}<0.2\\ a_{ij},a_{ij}\;⩾\;0.2 \end{array} \end{array}$$

Negative correlation was removed according to previous fMRI studies (Liao et al., 2013; Li et al., 2015). It was not calculated separately because the physiological basis of the negative correlations was ambiguous (Fox et al., 2009; Murphy et al., 2009).

ALFF measures the amplitude of time series fluctuation at each voxel (Zang et al., 2007), ReHo depicts the local synchronization of the time series of neighboring voxels (Zang et al., 2004), and DC represents the large-scale brain intrinsic connectivity in the voxel level (Buckner et al., 2009). Thus, the three measures of fMRI probe into the brain activity from different aspects.

### Statistical analysis

ALFF, ReHo, and DC maps of each frequency band were compared between the groups of children with ADHD and TDC. Two-sample *t*-tests were performed on the pooled data and each cohort, respectively. The full scale IQ and mean framewise displacement (FD) were included as nuisance covariates (Jenkinson et al., 2002; Yan et al., 2013), and cohort was further taken as a covariate for the *t*-tests on the pooled data. For each cohort, the statistical analyses were performed in study-specific functional volume masks including only voxels (in MNI152 standard space) present in at least 80% of the participants and then intersect with gray-matter mask to reduce non-cortical noise. The mask of the pooled data is the intersection of cohorts' masks. The results were corrected for multiple comparisons with a combined threshold of single voxel's *p* < 0.05 and cluster size > 139, 144, 136, 129, and 129 voxels for the cohorts and pooled data, corresponding to corrected *p* < 0.05 determined by Monte Carlo simulation and the mask of each cohort. The AlphaSim estimation was performed by DPABI V2.3 (http://rfmri.org/dpabi; Yan et al., 2016). At the same time, to reduce the possibility of false negative results and, hence, a more lenient threshold (*p* < 0.05, cluster size > 10 voxels) was also used for each cohort.

We also performed the analyses of standardized effect size (SES) of each measurement based on Cohen's d which is calculated as the equation as follows (Cohen, 2013):

(3) $$\begin{array}{l} \text{Cohen'}s\;d=\frac{{\bar{X}}_{ADHD}-{\bar{X}}_{TDC}}{S_{ALL}},\\ S_{ALL}=\sqrt{\frac{\left(n_{ADHD}-1\right)S_{ADHD}^{2}+\left(n_{TDC}-1\right)S_{TDC}^{2}}{n_{ADHD}+n_{TDC}-2}} \end{array}$$

According to equation of independent two-sample *t*-test as follows:

(4) $$\begin{array}{l} t=\frac{{\bar{X}}_{ADHD}-{\bar{X}}_{TDC}}{\sqrt{\frac{\left(n_{ADHD}-1\right)S_{ADHD}^{2}\;+\;\left(n_{TDC}-1\right)S_{TDC}^{2}}{n_{ADHD}\;+\;n_{TDC}-2}\left(\frac{1}{n_{ADHD}}\;+\;\frac{1}{n_{TDC}}\right)}} \end{array}$$

The relationship of Cohen's *d*- and *t*-value can be obtained as follows:

(5) $$\begin{array}{l} \text{Cohen'}s\text{ d}=t\sqrt{\frac{n_{ADHD}+n_{TDC}}{n_{ADHD}.n_{TDC}}} \end{array}$$

According to Equation (5), we transformed t maps into SES map for each cohort and pooled data. Then a combined threshold SES > 0.30 and cluster size > 129 voxels was used which corresponded to a combination threshold of *t* > 1.974 (*p* < 0.05) of the pooled data. The same threshold was applied to the SES maps of each cohort. SES of 0.30 corresponded to *t* = 1.141, 0.822, 1.124, and 0.926 (*p* = 0.26, 0.43, 0.27, and 0.36) for NYU, PKU1, PKU2, and PKU3, respectively.

To view the consistency of results, the thresholded *t*-maps and SES-maps were binarized and overlapped among the four cohorts. Further, in order to view how consistent the results of individual cohorts are with the pooled results, the overlapped map of cohorts was further overlapped with the binary map of the pooled data. The number of overlapped voxels across 4 and 3 cohorts was quantified using Dice overlap coefficient (Dice, 1944; Burunat et al., 2016) where the voxel number of intersection was divided by the total voxel number of all the cohorts.

## Results

### Results of pooled data in conventional frequency band

**Figure 2:**
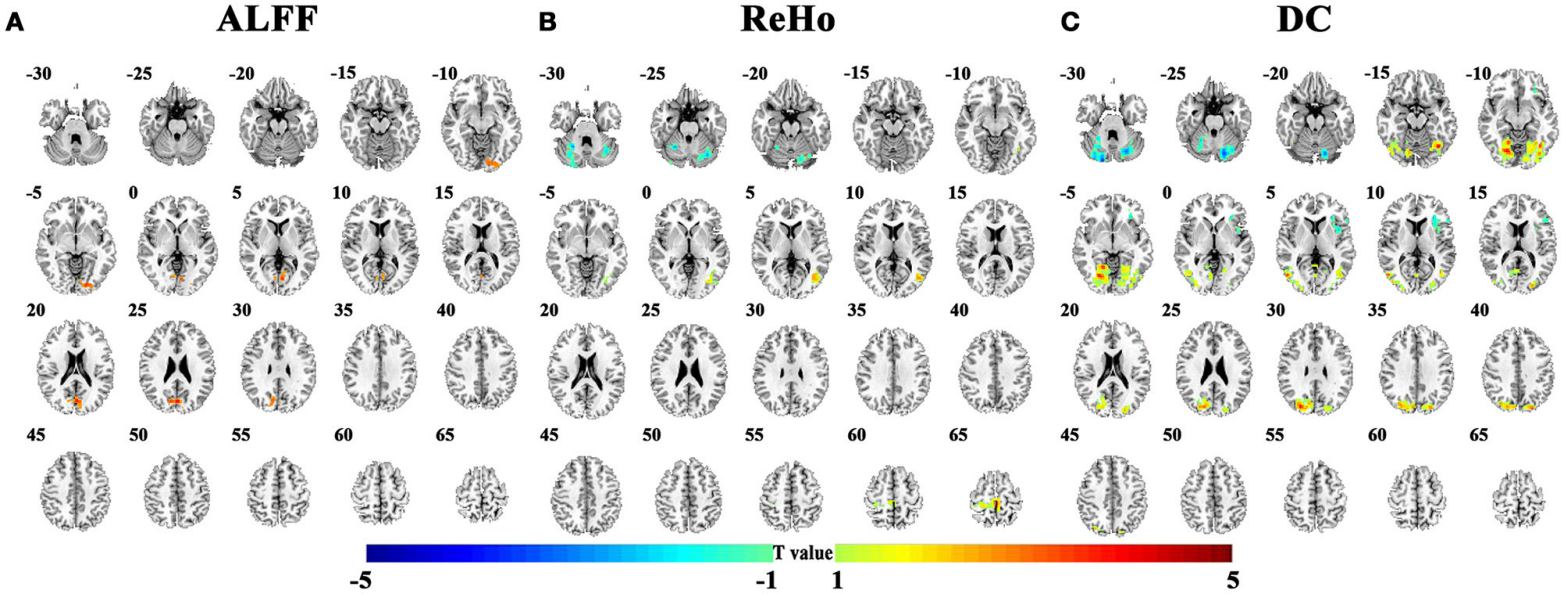
Differences of brain activity between TDC and children with ADHD on the pooled data. **(A–C)** indicate the results detected by ALFF (amplitude of low-frequency fluctuation), ReHo (regional homogeneity), and DC (degree centrality). The statistical threshold was set at *p* < 0.05, cluster size > 129 voxels, corresponding to corrected *p* < 0.05 determined by Monte Carlo simulation. Left in the figure indicates the right side of the brain.

The abnormal brain regions in the conventional low frequency band (0.01–0.08 Hz) for children with ADHD of the pooled data were shown in Figure 2 and Table 2. Children with ADHD had increased ALFF and DC in the bilateral lingual gyrus (Figures 2A,C). ReHo and DC were decreased in the bilateral cerebellum. In addition, the three methods detected some method-specific abnormality such as the bilateral paracentral lobule (Figure 2B) and the left insula (Figure 2C).

**Table 2:** Differences between TDC and ADHD on pooled data.

**Method**	**Region**	**L/R**	**BA**	**Peak MNI coordinates**			***t***	**Number of voxels**
				***x***	***y***	***z***		
ALFF	Lingual gyrus/Cuneus	L/R	18	0	−75	24	3.59	236
ReHo	Cerebellum	L/R	–	33	−69	−36	−3.70	537
	Paracentral lobule/Postcentral gyrus	L/R	3/4/6	−3	−33	69	4.15	187
	Mid. temporal gyrus	L	19	−6	51	−6	3.34	332
DC	Cerebellum	L/R	–	−18	−75	−24	−3.96	744
	Mid. occipital/Lingual gyrus	R	18	21	−72	−9	4.28	1,054
	Mid. occipital/Lingual gyrus	L	18	−39	−63	−12	4.30	635
	Insula	L	13	−30	30	9	−3.15	202

*ALFF, amplitude of low-frequency fluctuation; ReHo, regional homogeneity; DC, degree centrality; Mid., middle; L, left; R, right; BA, Brodmann's area*.

### Consistency across cohorts in conventional frequency band

**Figure 3:**
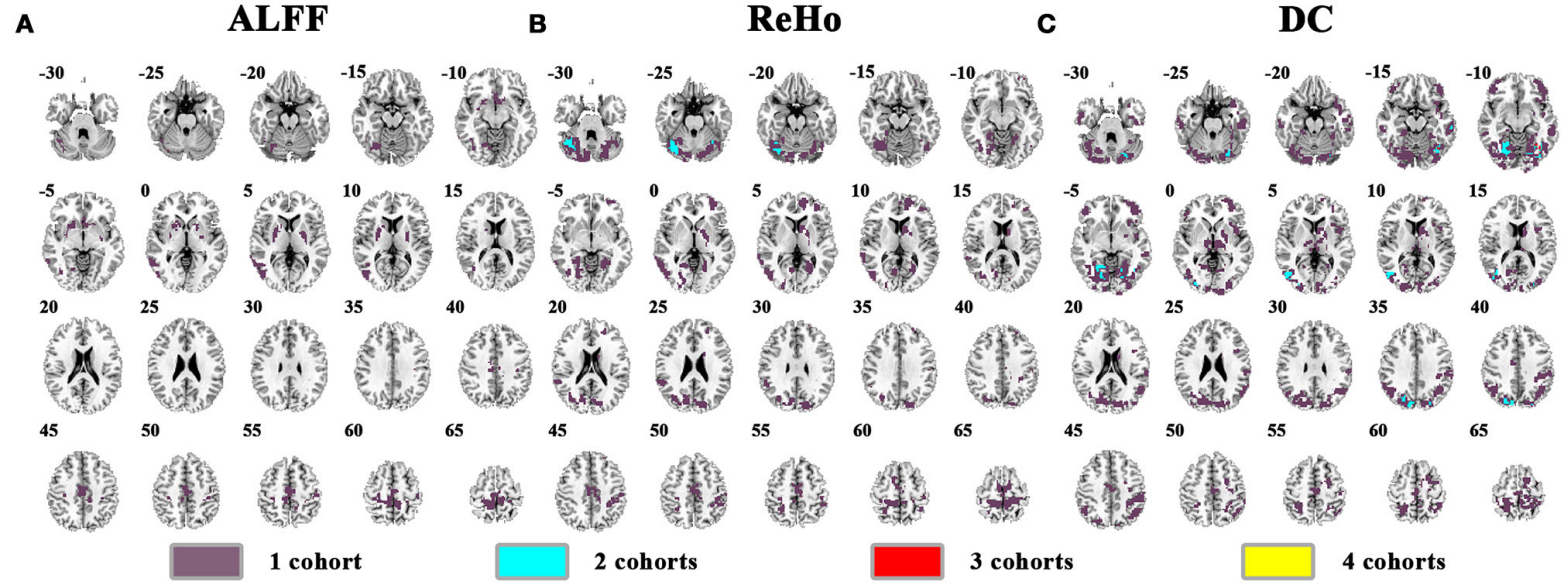
The overlapped results across the 4 cohorts. **(A–C)** indicate the results detected by ALFF, ReHo, and DC, respectively. Purple indicates the regions detected in only one of the 4 cohorts. Mint, red, and yellow indicate the regions detected in 2, 3, and 4 cohorts, respectively.

The abnormal brain activity in the conventional frequency band (0.01–0.08 Hz) was identified for each cohort, and the overlapped results across the four cohorts were shown in Figure 3 (See details of each cohort in Supplementary Figures 1–6). Only a few voxels showed overlapped abnormality from three or four cohorts by any method (ALFF, ReHo, or DC). Using DC, we observed 6 voxels overlapped from NYU, PKU2, and PKU3 in the left inferior occipital gyrus and fusiform gyrus. Even if taking the overlapped abnormality from 2 cohorts into consideration, only a few clusters were overlapped, e.g., the cerebellum by ReHo as well by DC (Figures 3B,C), the bilateral cuneus (Figure 3C) by DC.

**Figure 4:**
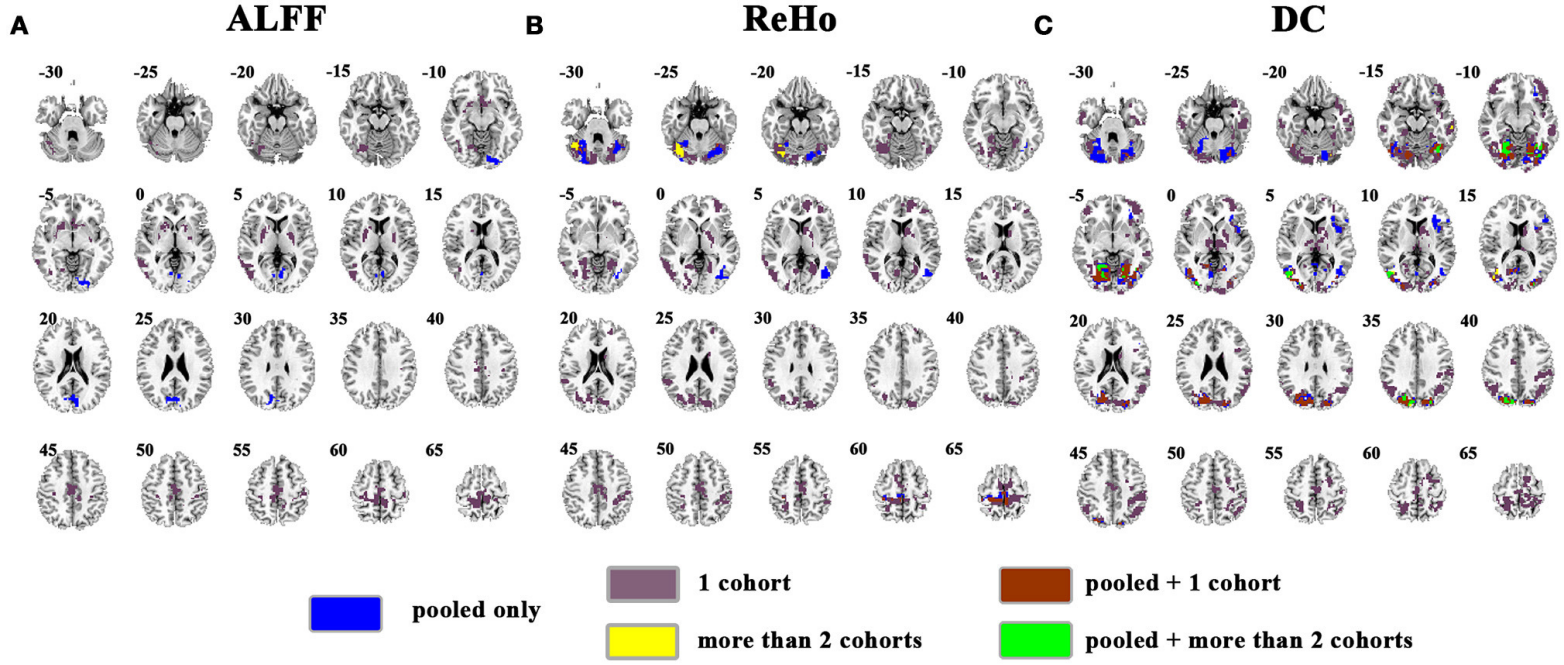
Overlapped results of the pooled data and individual cohorts. **(A–C)** indicate the results detected by ALFF, ReHo, and DC, respectively. Blue indicates the regions detected only in pooled data. Purple indicates the regions detected only in one of the 4 cohorts. Yellow indicates the regions detected by only 2 of the 4 cohorts but not in the pooled data. Brown indicates the regions detected in the pooled data and in only one of the 4 cohorts. Green indicates the regions detected in the pooled data and 2 of the 4 cohorts. Left in the figure indicates the right side of the brain.

The overlapped results of the pooled data and individual cohorts were shown in Figure 4. Some clusters detected in individual cohorts could not be observed in the results of pooled data, e.g., in the cuneus for ReHo (purple marked in Figure 4B) and thalamus for DC (purple marked in Figure 4C). Although, some clusters could be identified as the overlapped regions from two cohorts, they were not be observed in the pooled data, such as the right cerebellum for ReHo (yellow marked in Figure 4B).

**Figure 5:**
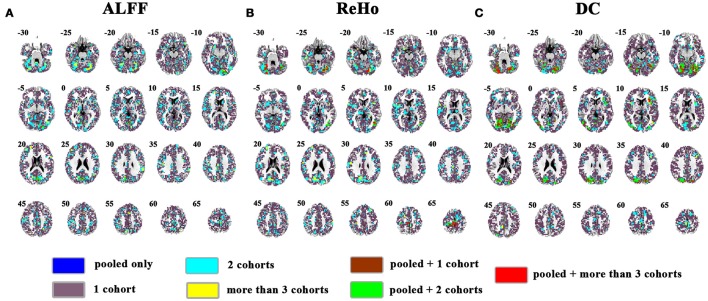
Overlapped effect size results of the pooled data and individual cohorts. The threshold of effect size was set at 0.3 for the pooled data and each cohort. **(A–C)** indicate the results detected by ALFF, ReHo, and DC, respectively. Blue indicates the regions detected only in pooled data. Purple indicates the regions detected only in one of the 4 cohorts. Mint indicates the regions detected in 2 cohorts. Yellow indicates the regions detected by only 3 or 4 cohorts but not in the pooled data. Brown indicates the regions detected in the pooled data and in only one cohort. Green indicates the regions detected in the pooled data and 2 cohorts. Red indicates the regions detected in the pooled data and 3 or 4 cohorts. Left in the figure indicates the right side of the brain.

**Figure 6:**
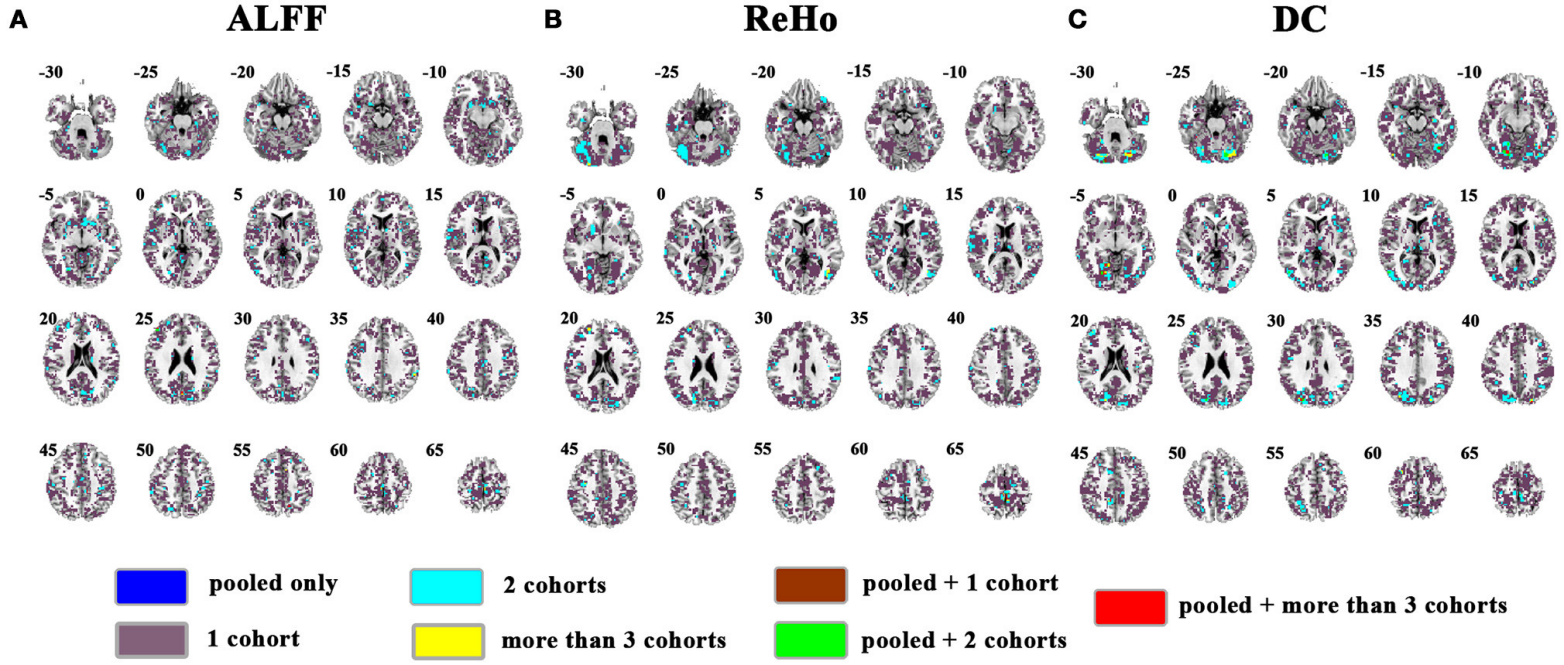
Overlapped effect size results of the pooled data and individual cohorts. The threshold of effect size was set at 0.5 for the pooled data and each cohort. **(A–C)** indicate the results detected by ALFF, ReHo, and DC, respectively. Blue indicates the regions detected only in pooled data. Purple indicates the regions detected only in one of the 4 cohorts. Mint indicates the regions detected in 2 cohorts. Yellow indicates the regions detected by only 3 or 4 cohorts but not in the pooled data. Brown indicates the regions detected in the pooled data and in only one cohort. Green indicates the regions detected in the pooled data and 2 cohorts. Red indicates the regions detected in the pooled data and 3 or 4 cohorts. Left in the figure indicates the right side of the brain.

The overlapped SES maps of each cohort and pooled data for ALFF, ReHo, and DC were shown in Figure 5 (with a combined threshold of SES > 0.3). Some clusters showing overlaps from more than 3 cohorts could be also shown in the pooled data (red marked). These clusters included the bilateral cerebellum for ReHo and DC (Figures 5B,C), right calcarine for ALFF and DC (Figures 5A,C) and the bilateral paracentral lobule for ReHo (Figure 5B). However, if the SES threshold was set at 0.5, these clusters showed no overlap (Figure 6). The overlapped SES maps across the 4 cohorts and the SES maps of the pooled data were shown in Supplementary Figure 7.

### Consistency across cohorts in sub-frequency bands

After investigation in conventional low frequency band (0.01–0.08 Hz) as shown above, overlapped results across cohort were further examined in several sub-frequency bands including Slow-6/5/4/3/2. Furthermore, to reduce the possibility of false negative results and, a more lenient threshold (*p* < 0.05, cluster size > 10 voxels) was also applied for each cohort. There is no voxel overlapped by all the cohorts. The number of the overlapped voxels was not more than 12 across three cohorts, and the highest Dice overlap coefficient is only 0.0131 (Table 3). In each sub-frequency band, most overlapped clusters were also observed from 2 cohorts (see details in Supplementary Figures 8–10).

**Table 3:** Clusters which were the overlap for three/four cohorts and contained maximal number of voxels.

**Method**	**Number of overlapped cohorts**	**Region**	**L/R**	**BA**	**Number of overlapped voxels**	**Dice**
**CONVENTIONAL BAND (0.01–0.08 Hz)**						
ALFF	4			None		
	3			None		
ReHo	4			None		
	3	Paracentral lobule	R	4	12	0.0041
DC	4			None		
	3	Cerebellum	R	–	12	0.0096
**SLOW-6 (0–0.01 Hz)**						
ALFF	4			None		
	3			None		
ReHo	4			None		
	3	Med. frontal cortex	L	11	1	0.0004
DC	4			None		
	3			None		
**SLOW-5 (0.01–0.027 Hz)**						
ALFF	4			None		
	3			None		
ReHo	4			None		
	3	Paracentral lobule	L/R	4	3	0.0016
DC	4			None		
	3	Paracentral lobule	L/R	4	7	0.0025
**SLOW-4 (0.027–0.073 Hz)**						
ALFF	4			None		
	3			None		
ReHo	4			None		
	3	Cerebellum	R	–	6	0.0026
DC	4			None		
	3	Mid. occipital gyrus	L	19	11	0.0131
**SLOW-3 (0.073–0.198 Hz)**						
ALFF	4			None		
	3	Paracentral lobule	L	4	1	0.0005
ReHo	4			None		
	3	Mid. frontal gyrus	L	8	2	0.0013
DC	4			None		
	3	Supplementary motor area	R	6	1	0.0004
**SLOW-2 (0.198–0.25 Hz)**						
ALFF	4			None		
	3			None		
ReHo	4			None		
	3	Sup. frontal gyrus	R	6	2	0.001
DC	4			None		
	3			None		

*Sup., superior; Mid., Middle; Med., Medial; L, left; R, right. The threshold was p < 0.05 and cluster size > 10 voxels for each cohort*.

## Discussion

The present study examined the consistency of abnormal local brain activity across cohorts of ADHD-200. We applied three voxel-wise whole brain analytic methods (ALFF, ReHo, and DC), strict and lenient statistical thresholds, and conventional frequency band (0.01–0.08 Hz) and sub-frequency bands (Slow/2/3/4/5/6) in the analysis process. Results from these analyses indicated that the abnormal local brain activity across cohorts of ADHD-200 was inconsistent.

The data of all four cohorts were first pooled together in the present study, as the general process way of the studies using ADHD-200 (Sato et al., 2013; Zhang et al., 2014). The abnormal brain activity for ADHD was identified in the clusters, such as the bilateral sensorimotor cortices and the bilateral lingual gyrus. Our further analysis showed that these results from pooled data were not consistent across cohorts. Most of the clusters identified for pooled data could not be observed in the results for individual cohort. This finding was further supported by the analyses of SES. The overlapped regions did not reach a medium (0.5) level. Thus, the results of directly pooled data from different cohorts do not mean consistent results among the cohorts included, and the SES of the results should be examined in the future studies of large sample dataset. Future studies derived from multi-site large-sample dataset should not only present the statistical result of a pooled data, but also present the results of each cohort of both *t*-map and SES.

Moreover, all examined cohorts did not exhibit overlapped clusters, suggesting a high heterogeneity of ADHD. We noticed a recent finding that detected the consistent abnormality across cohorts of ADHD-200 (Cai et al., 2015). Using the resource allocation index (RAI) (a measure of network interactions across the SN, CEN, and DMN), Cai et al. found RAI was significantly lower in children with ADHD than in control subjects and the results were reproducible across three independent cohorts. While abnormality of network interaction may reveal the complexity of spontaneous brain activity in ADHD, it could not illustrate which brain region is abnormal. From the perspective of clinical practice, analytic methods for precise localization of the abnormality in a whole-brain voxel-based way should be emphasized. Whole-brain voxel-based analysis facilities coordinate-based meta-analysis (CB-meta) which can help to define precise localization of abnormal spontaneous brain activity by quantitatively aggregating independent results reported in a standard coordinate space (Eickhoff et al., 2009) and further help to guide intervention therapies, such as deep brain stimulation and transcranial magnetic stimulation (Zang et al., 2015). Thus, the present study used three whole-brain voxel-based measurements, i.e., ALFF, ReHo, and DC. These measurements are widely employed in rs-fMRI studies to access local brain activity from different aspects. Here we applied these three measurements to explore the consistent local abnormality of children with ADHD across cohorts. Nevertheless, consistent results across cohorts were not identified through any one of the three measurements.

The present study not only focused on the conventional frequency band but also stressed several sub-frequency bands. Frequency-dependent investigation provides us a new prospect to investigate the physiological mechanism of the brain activity. A recent rs-fMRI study reported some frequency-dependent abnormalities for children with ADHD (Yu et al., 2015). For example, in the orbital frontal cortex (OFC), the frequency bands of slow-3 and slow-2 contributed more to the differences than did the slow-5 and slow-4 bands. We found that the detected differences between ADHD and TDC are different according to different frequency bands. For example, compared with TDC, children with ADHD had decreased DC in the left inferior parietal gyrus only in slow-3 but others frequency bands and decreased DC in the bilateral putamen/thalamus only in slow-4 but others frequency bands (Supplementary Figure 11). Previous studies often consider the Slow-6 (<0.01 Hz) as signal drift, and it was usually discarded from further analysis. However, our recent publications on finger force feedback task have challenged this issue. ReHo and ALFF of basal ganglia in Slow-6 showed difference between real and sham feedback conditions, and the ALFF in Slow-6 was related to finger force (Zhang et al., 2015a,b). Moreover, ReHo difference between ADHD and TDC in Slow-6 was detected in previous study (Yu et al., 2015). Thus, the Slow-6 was involved in our analysis and differences can be detected. However, the results couldn't be detected in any other cohorts.

Several limitations exist in the present study. First, we could not explore the contribution of different subtype to the inconsistency in ADHD neuroimaging findings because of the small sample size for statistical analysis. For example, PKU1 only included 6 inattention and 9 combined subjects. Their contribution should be explored on a large sample dataset in the future. Second, we only used three whole brain voxel-based measurements to evaluate the consistency across cohorts. Thus, our observations were restricted to these measurements. Investigations with more whole-brain voxel-based measurements will be helpful.

## Conclusions

Data-sharing projects like ADHD-200 provide large sample analysis. But pooled data itself is not enough. The current study used three whole-brain voxel-based analytic methods, i.e., ALFF, ReHo, and DC not only on the pooled data but also on each individual cohort. We found that the findings based on the pooled data of ADHD-200 were inconsistent across the individual cohorts. Even in a more lenient threshold, this inconsistency could be observed. Such inconsistency could be found not only in the conventional low frequency-band (0.01–0.08 Hz) but also in a few sub-frequency band of Slow-2/3/4/5/6. These results support the view that ADHD is a highly heterogeneous disorder. Future studies should try more efforts on exploring more consistent findings of rs-fMRI data of ADHD. Data sharing could benefit improving the reproducibility of neuroimage studies, and we suggest that analysis based on multi-site large-sample dataset should be performed on pooled data and single cohort, respectively.

## Author contributions

YZ, HZ, and QC conceived and designed the experiment. JW and HZ performed the data analysis. YZ, LZ, LS, and YW provided advice on the analysis and interpretation of the results. JW, HZ, and YZ wrote the paper.

### Conflict of interest statement

The authors declare that the research was conducted in the absence of any commercial or financial relationships that could be construed as a potential conflict of interest.
