# Supplementary material for: Inconsistency in Abnormal Brain Activity across Cohorts of ADHD-200 in Children with Attention Deficit Hyperactivity Disorder
Source: Front Neurosci. 2017 Jun 6;11:320. doi: 10.3389/fnins.2017.00320 (PMC5459906; doi:10.3389/fnins.2017.00320)
Supplement: Supplementary file 1 [file Presentation1.PDF]

Supplementary Material

Inconsistency in abnormal brain activity across cohorts of ADHD-200 in children with attention deficit hyperactivity disorder

Jian-Bao Wang[1,2,3], Li-Jun Zheng[1,2,3], Qing-Jiu Cao[4], Yu-Feng Wang[4], Li Sun[4], Yu-Feng Zang[1,2,3*], Hang Zhang[5*]

1. Center for Cognition and Brain Disorders and the Affiliated Hospital, Hangzhou Normal University, Hangzhou, China

2. Zhejiang Key Laboratory for Research in Assessment of Cognitive Impairments, Hangzhou, China

3. Institutes of Psychological Sciences, Hangzhou Normal University, Hangzhou, China

4. Institute of Mental Health, Peking University, Beijing, China

5. Paul C. Lauterbur Research Centers for Biomedical Imaging, Shenzhen Institutes of Advanced Technology, Chinese Academy of Sciences, Shenzhen 518055, China.

*: Corresponding to kevinhangbnu@foxmail.com (Hang Zhang) or zangyf@gmail.com (ZANG Yu-Feng).

1. Supplementary Figures and Tables

1.1 Supplementary Table

Supplementary Table 1. Functional parameters

sites	TR/TE/Flip Angle	Slice thickness/Gap	FOV	Slice	TP	duration
**NYU**	2000 ms/15 ms/90 °	4.0 mm/0mm	240×192mm	33	176	6 min
**PKU1**	2000 ms/30 ms/90 °	3.5 mm/0.7mm	200×200mm	33	236	8 min
**PKU2**	2000 ms/30 ms/90 °	3.0 mm/0.6mm	200×200mm	33	236	8 min
**PKU3**	2000 ms/30 ms/90 °	4.5 mm/0mm	220×220mm	30	236	8 min

The parameters of T1-weighted structural image can be found in http://fcon_1000.projects.nitrc.org/indi/adhd200/.

1.2 Supplementary Figures

1.2.1 Results of each cohort in conventional frequency band (0.01-0.08 Hz)

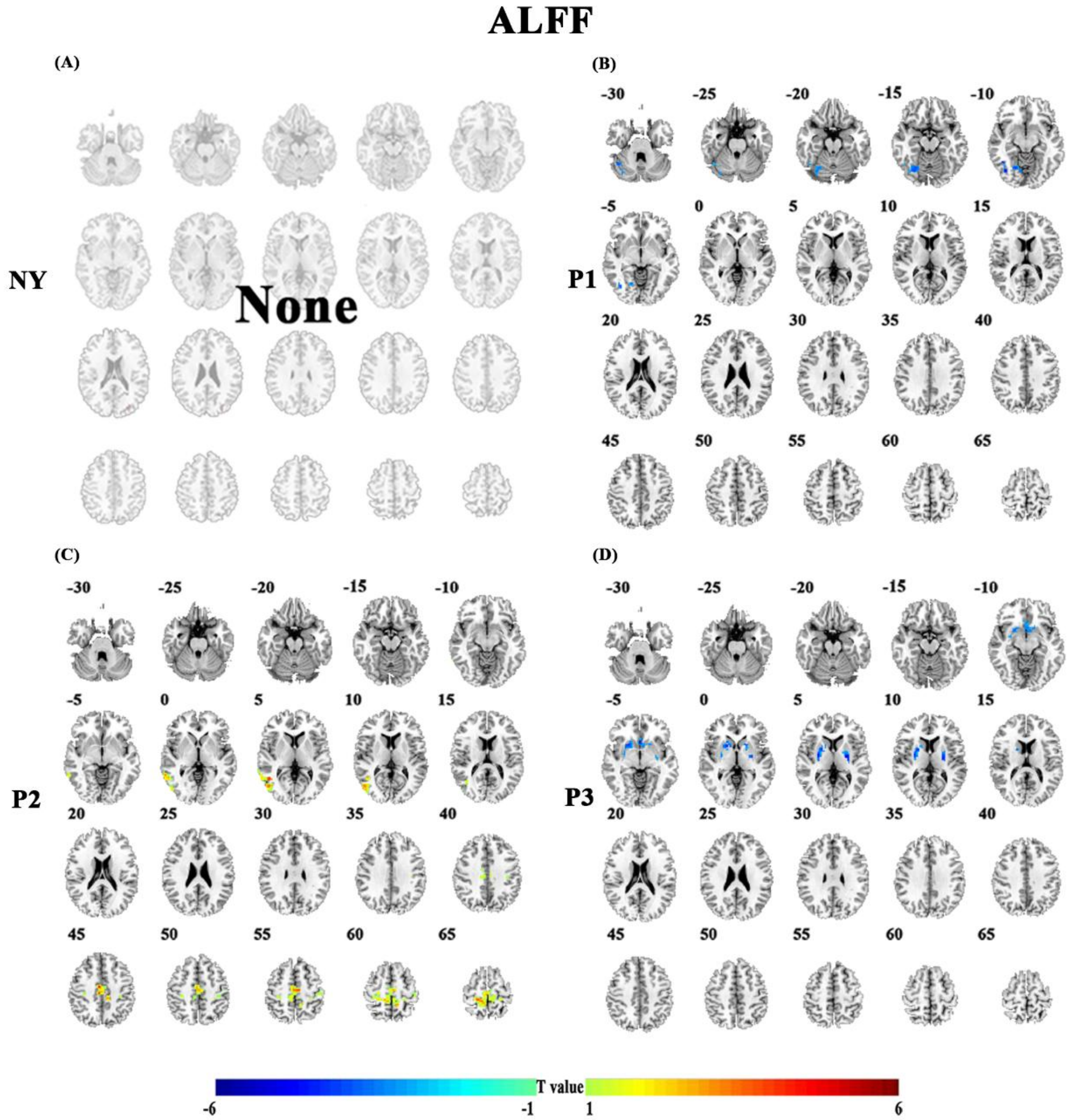

Supplementary Figure 1. Clusters showing significant ALFF differences between TDC and children with ADHD. (A), (B), (C) and (D) indicate the result of NYU, PKU1, PKU2 and PKU3, respectively. Cold color indicates the region in which ADHD subjects had decreased activity compared with TDC and the warm color indicates the opposite.

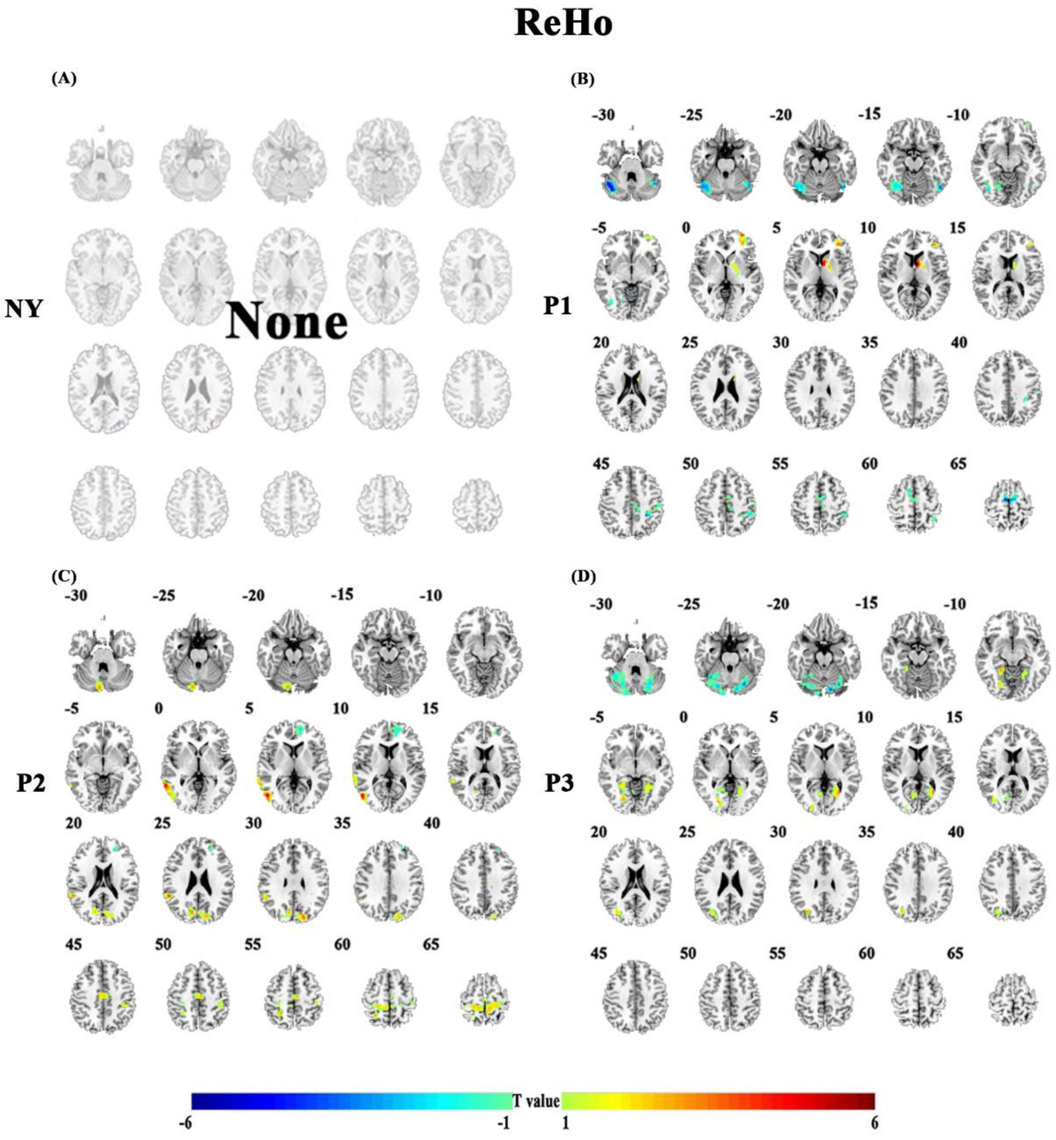

Supplementary Figure 2. Clusters showing significant ReHo differences between TDC and children with ADHD. (A), (B), (C) and (D) indicate the result of NYU, PKU1, PKU2 and PKU3, respectively. Cold color indicates the region in which ADHD subjects had decreased activity compared with TDC and the warm color indicates the opposite.

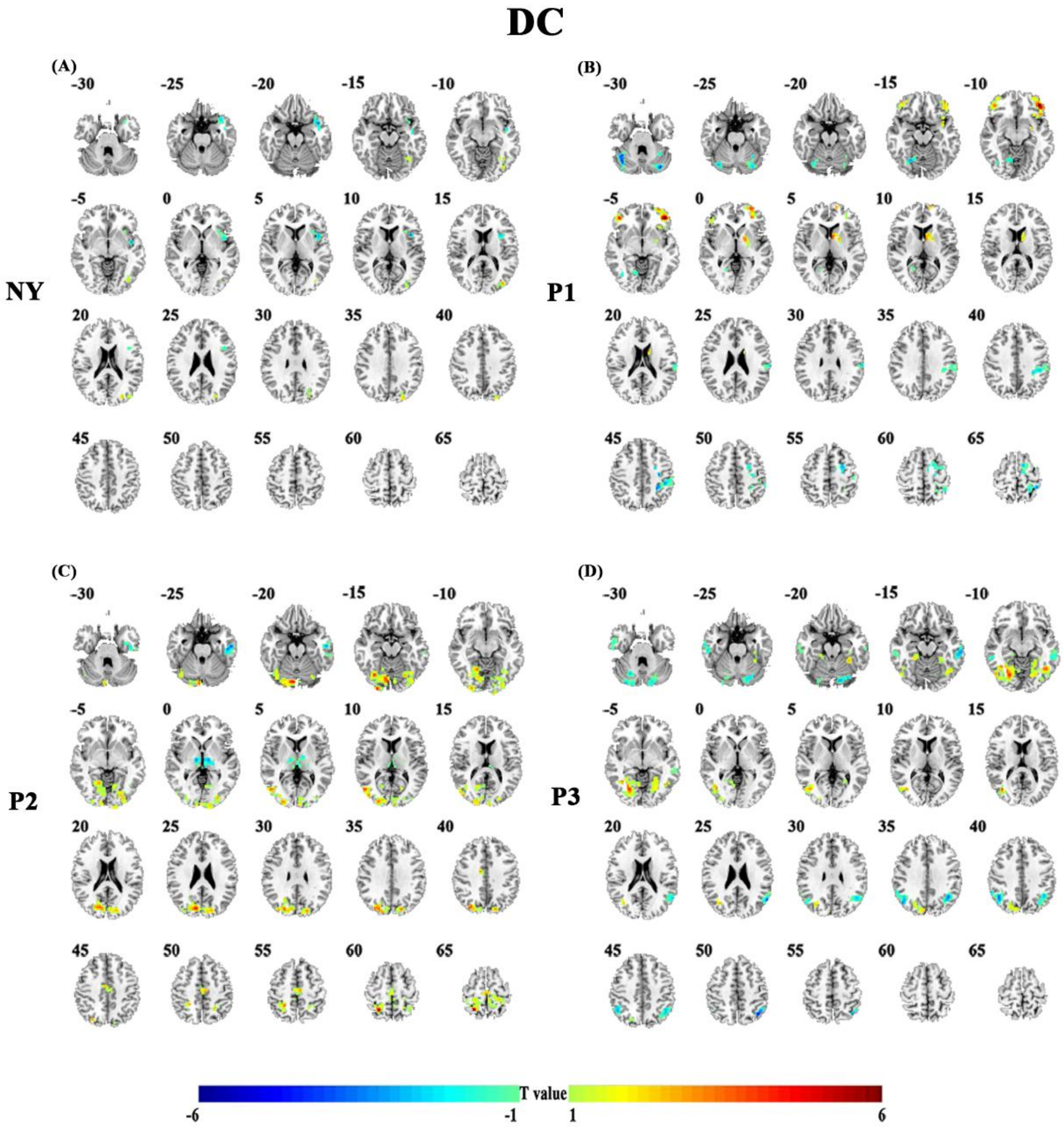

Supplementary Figure 3. Clusters showing significant DC differences between TDC and children with ADHD. (A), (B), (C) and (D) indicate the result of NYU, PKU1, PKU2 and PKU3, respectively. Cold color indicates the region in which ADHD subjects had decreased activity compared with TDC and the warm color indicates the opposite.

1.2.2 Results of each cohort in conventional frequency band (0.01-0.08 Hz) in lenient threshold

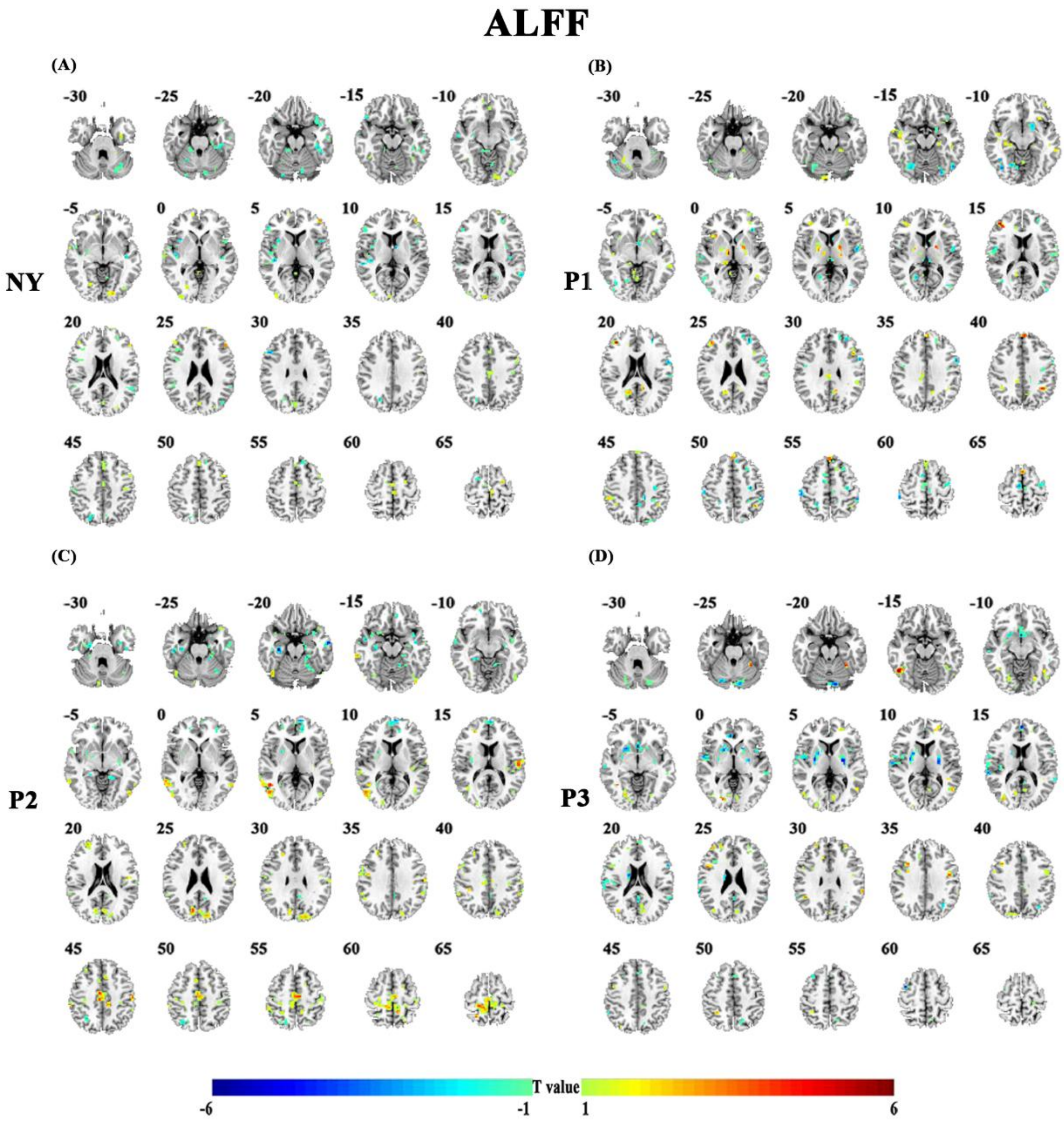

Supplementary Figure 4. Clusters showing significant ALFF differences between TDC and children with ADHD at a lenient threshold ($p < 0.05$, cluster size > 10 voxels) in each cohort. (A), (B), (C) and (D) indicate the result of NYU, PKU1, PKU2 and PKU3, respectively. Cold color indicates the region in which ADHD subjects had decreased activity compared with TDC and the warm color indicates the opposite.

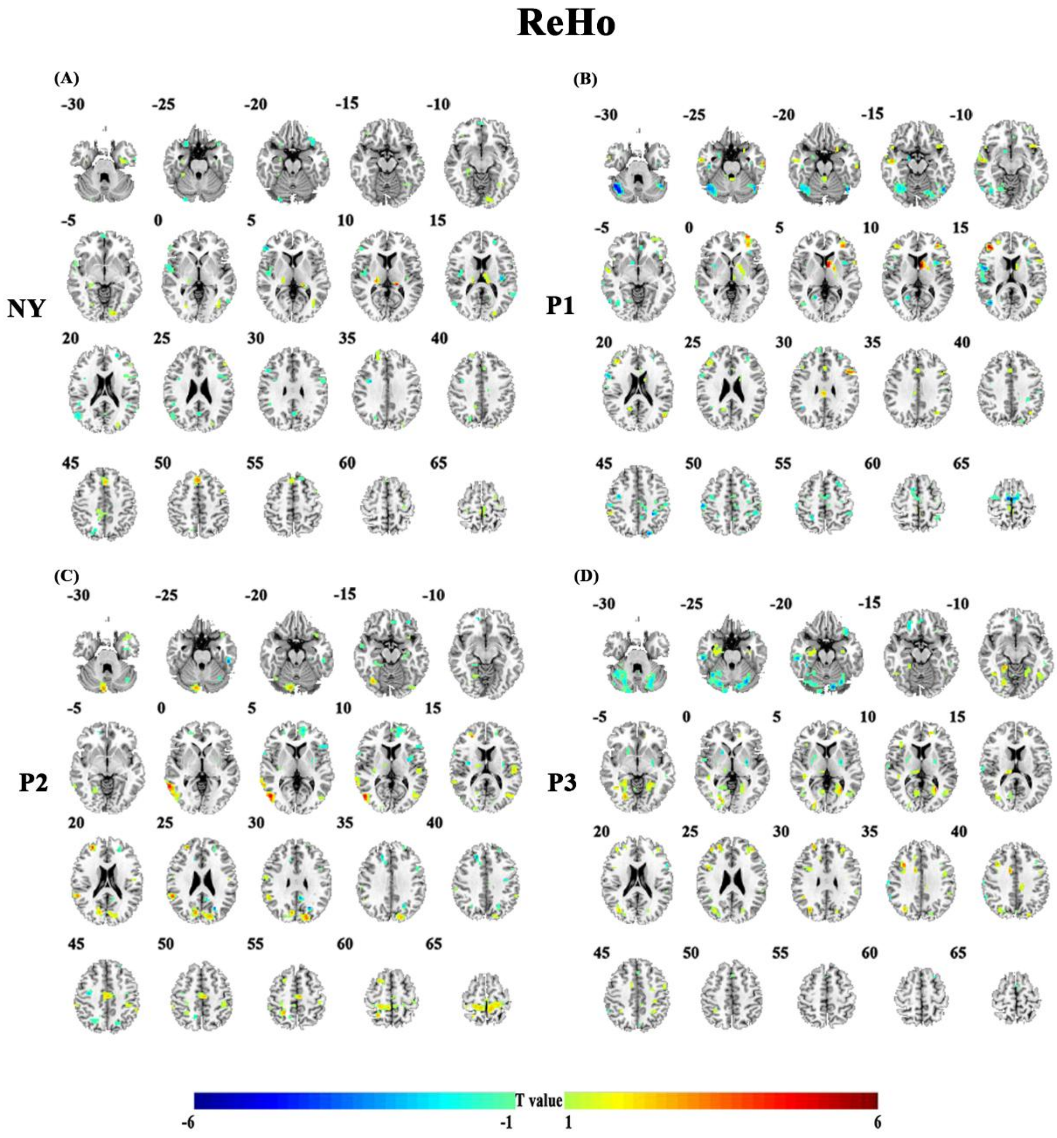

Supplementary Figure 5. Clusters showing significant ReHo differences between TDC and children with ADHD at a lenient threshold ($p < 0.05$, cluster size > 10 voxels) in each cohort. (A), (B), (C) and (D) indicate the result of NYU, PKU1, PKU2 and PKU3, respectively. Cold color indicates the region in which ADHD subjects had decreased activity compared with TDC and the warm color indicates the opposite.

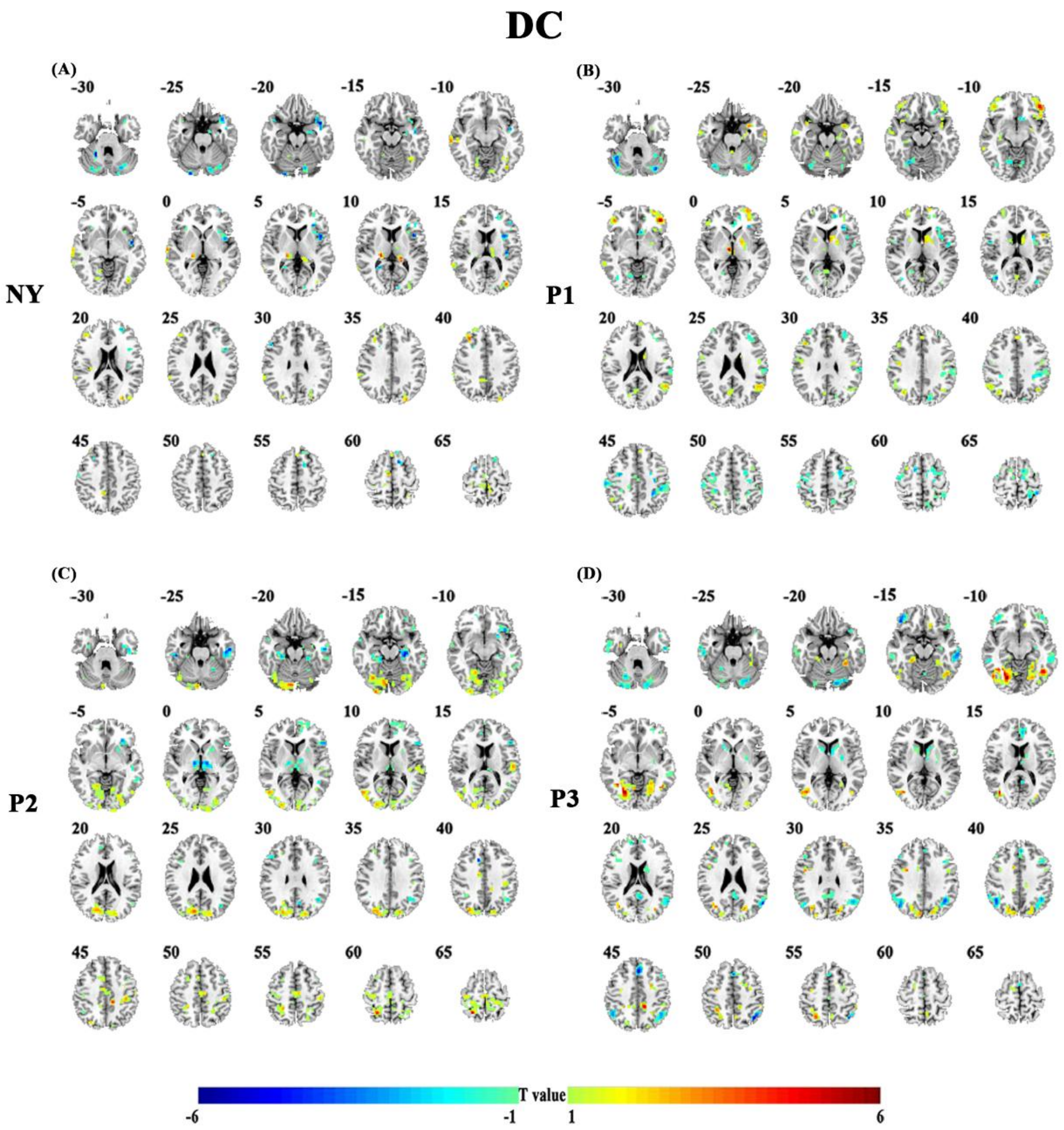

Supplementary Figure 6. Clusters showing significant DC differences between TDC and children with ADHD at a lenient threshold ($p < 0.05$, cluster size > 10 voxels) in each cohort. (A), (B), (C) and (D) indicate the result of NYU, PKU1, PKU2 and PKU3, respectively. Cold color indicates the region in which ADHD subjects had decreased activity compared with TDC and the warm color indicates the opposite.

1.2.3 Overlapping of effect size across each cohort and pooled data

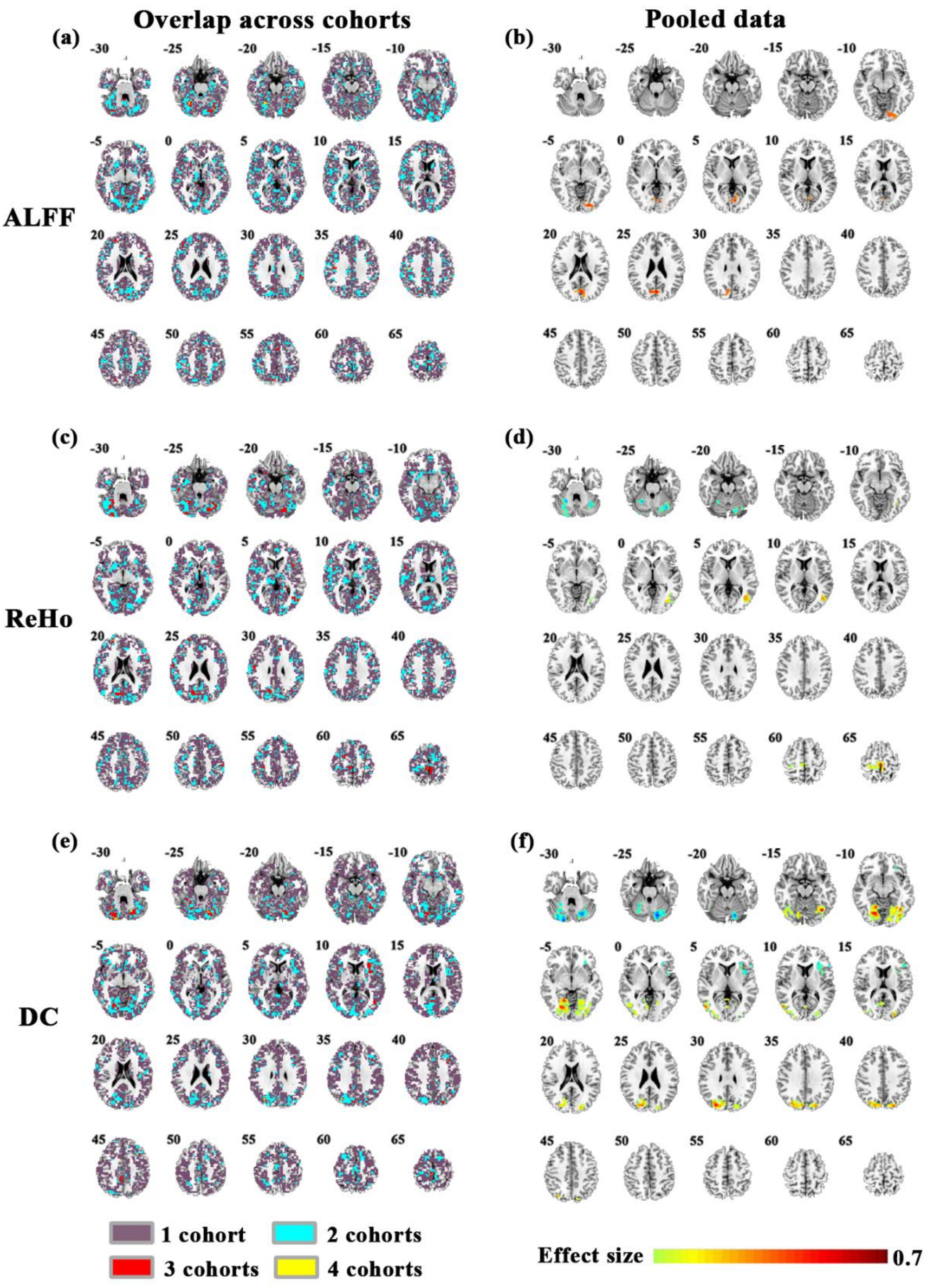

Supplementary Figure 7. The overlapped effect size across the 4 cohorts and the effect size of pooled data for ALFF, ReHo and DC. Mint, red, and yellow indicate the

regions detected in 2, 3, and 4 cohorts, respectively. In each cohort, the threshold was set at ES > 0.30.

1.2.4 Overlapping across each cohort in each frequency band

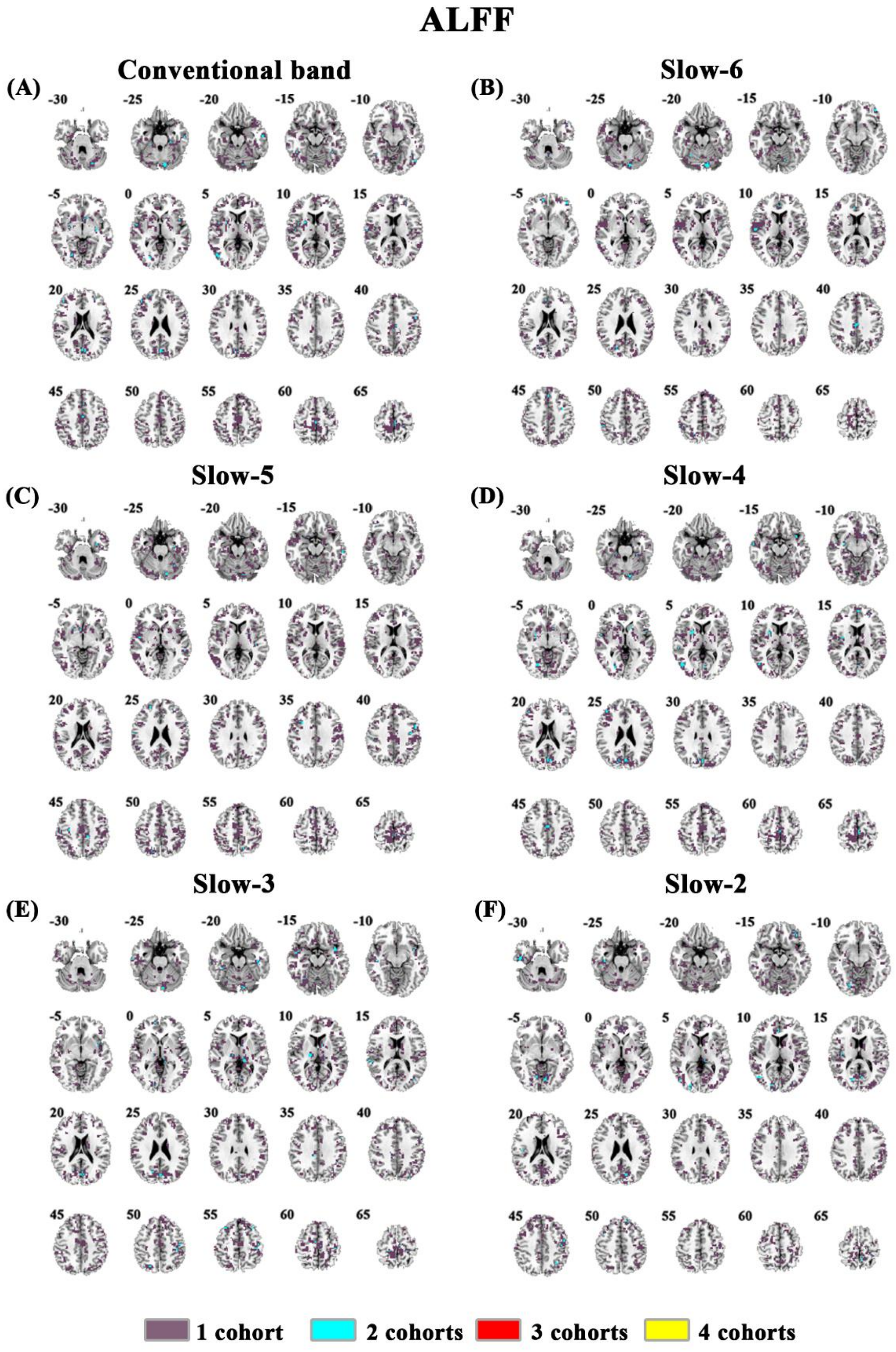

Supplementary Figure 8. The overlapped results of ALFF across the 4 cohorts. (A) to (F)

indicate each frequency band. Purple indicates the regions detected in only one of the 4 cohorts. Mint, red, and yellow indicate the regions detected in 2, 3, and 4 cohorts, respectively. In each cohort, the statistical threshold was set at $p < 0.05$, cluster size > 10 voxels.

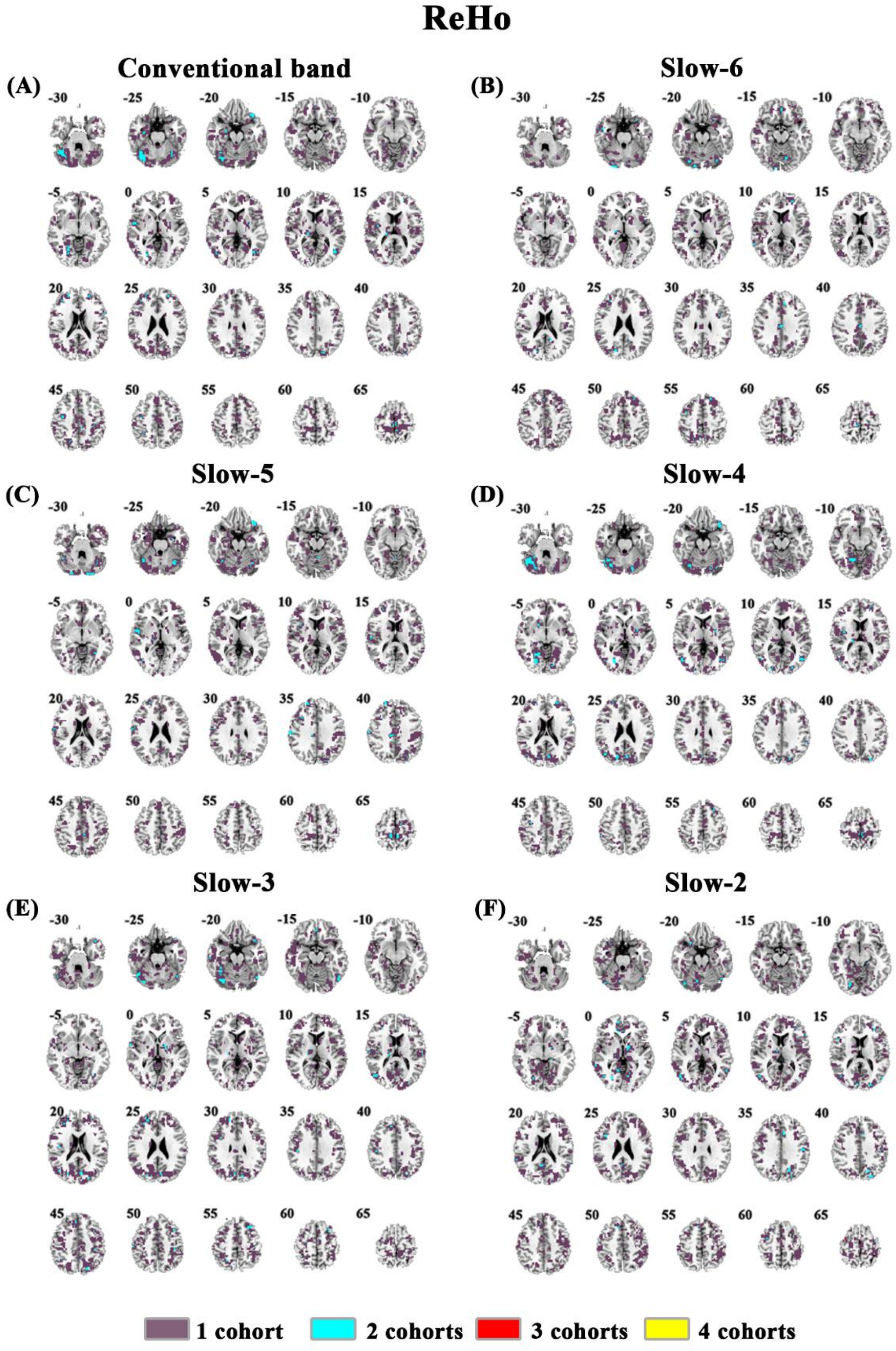

Supplementary Figure 9. The overlapped results of ReHo across the 4 cohorts. (A) to (F)

indicate each frequency band. Purple indicates the regions detected in only one of the 4 cohorts. Mint, red, and yellow indicate the regions detected in 2, 3, and 4 cohorts, respectively. In each cohort, the statistical threshold was set at $p < 0.05$, cluster size > 10 voxels.

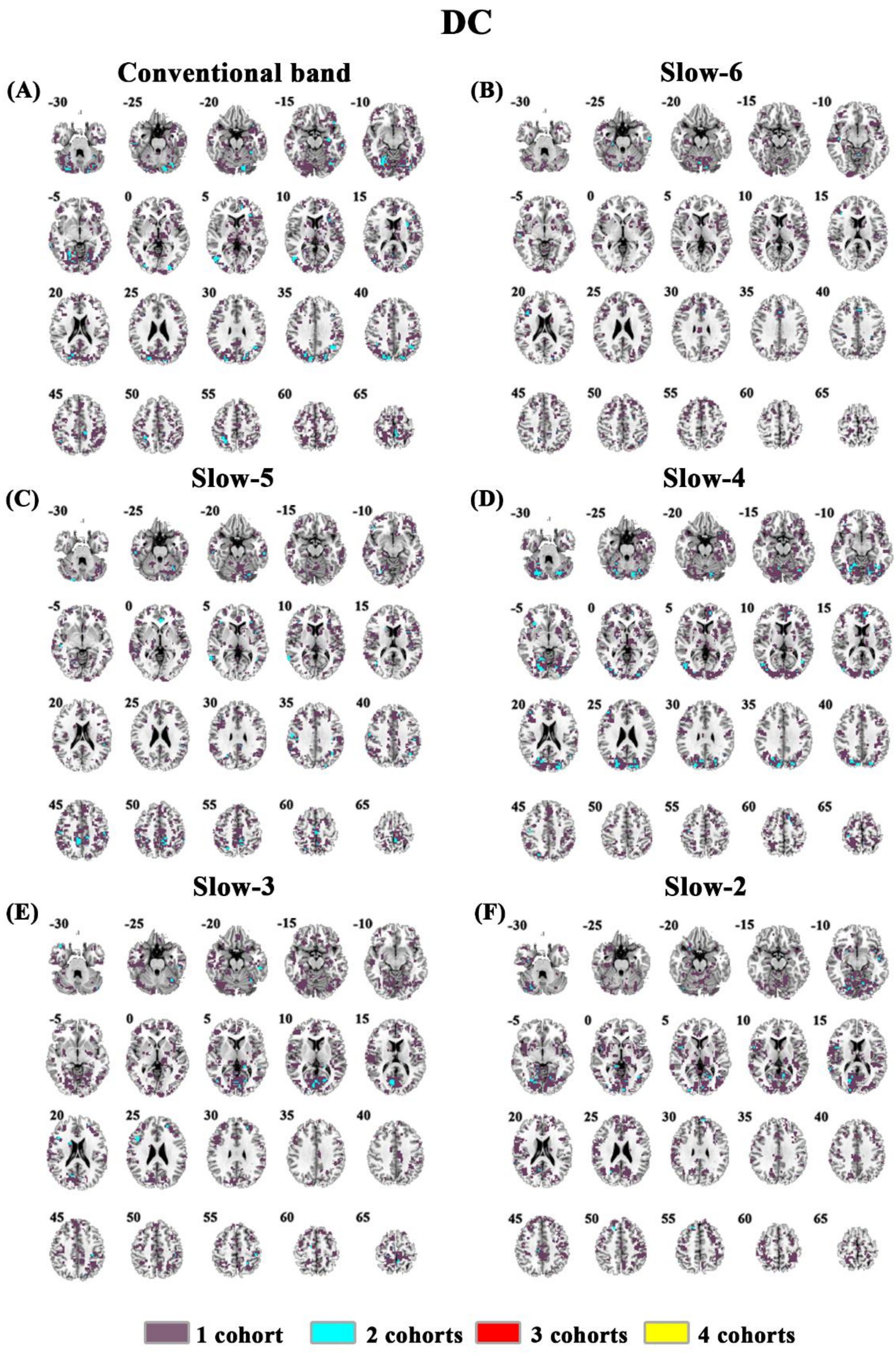

Supplementary Figure 10. The overlapped results of DC across the 4 cohorts. (A) to (F) indicate each frequency band. Purple indicates the regions detected in only one of the 4 cohorts. Mint, red, and yellow indicate the regions detected in 2, 3, and 4 cohorts, respectively. In each cohort, the statistical threshold was set at $p < 0.05$, cluster size > 10 voxels.

1.2.5 Example for the frequency-dependent feature

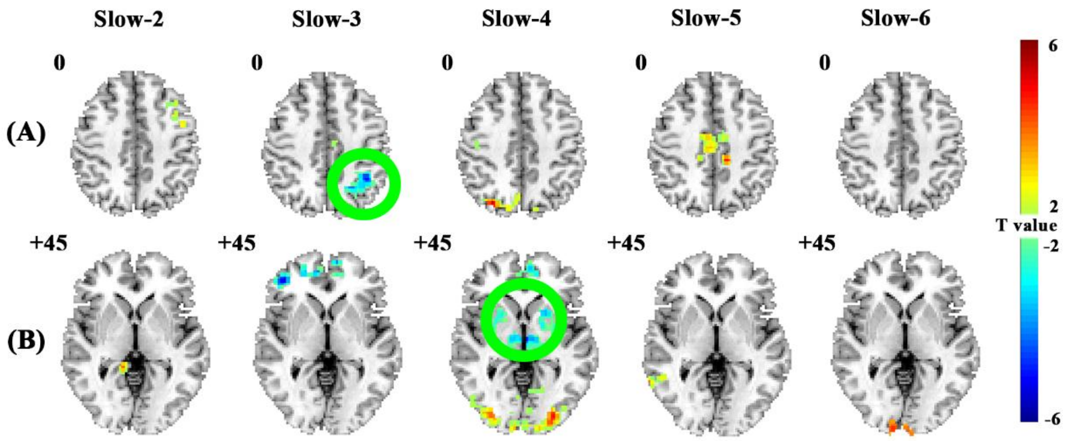

Supplementary Figure 11. Clusters showing frequency-dependent differences of DC between TDC and ADHD for PKU2. Cold color indicates the region in which ADHD subjects had decreased activity compared with TDC and the warm color indicates the opposite. The statistical threshold was set at $p < 0.05$, cluster size > 132 voxels.
